# Food supply confers calcifiers resistance to ocean acidification

**DOI:** 10.1038/srep19374

**Published:** 2016-01-18

**Authors:** Laura Ramajo, Elia Pérez-León, Iris E. Hendriks, Núria Marbà, Dorte Krause-Jensen, Mikael K. Sejr, Martin E. Blicher, Nelson A. Lagos, Ylva S. Olsen, Carlos M. Duarte

**Affiliations:** 1 Global Change Department, Instituto Mediterráneo de Estudios Avanzados (IMEDEA, CSIC-UIB), C/ Miquel Marqués 21, 07190 Esporles, Islas Baleares, Spain; 2 Centro de Investigación e Innovación para el Cambio Climático (CiiCC), Universidad Santo Tomás, Avda. Ejército 146, 8370003 Santiago, Chile; 3 Department of Bioscience, Aarhus University, Vejlsøvej 25, 8600 Silkeborg, Denmark; 4 Arctic Research Centre, Bioscience, Aarhus University, C. F. Møllers Allé 8, 8000 Aarhus, Denmark; 5 Greenland Climate Research Centre, Greenland Institute of Natural Resources, Kivioq 2 P.O. Box 570, 3900 Nuuk, Greenland; 6 Plant Biology and The UWA Oceans Institute, University of Western Australia, 35 Stirling Highway, Crawley, WA 6009, Australia; 7 King Abdullah University of Science and Technology (KAUST), Red Sea Research Center (RSRC), Thuwal, 23955-6900, Saudi Arabia

## Abstract

Invasion of ocean surface waters by anthropogenic CO_2_ emitted to the atmosphere is expected to reduce surface seawater pH to 7.8 by the end of this century compromising marine calcifiers. A broad range of biological and mineralogical mechanisms allow marine calcifiers to cope with ocean acidification, however these mechanisms are energetically demanding which affect other biological processes (trade-offs) with important implications for the resilience of the organisms against stressful conditions. Hence, food availability may play a critical role in determining the resistance of calcifiers to OA. Here we show, based on a meta-analysis of existing experimental results assessing the role of food supply in the response of organisms to OA, that food supply consistently confers calcifiers resistance to ocean acidification.

Increased CO_2_ derived from anthropogenic emissions is causing a decrease in surface ocean pH^1^,^2^,^3^, carbonate ion concentration ([CO_3_^2−^]) and saturation states of calcite and aragonite^3^, which are predicted to affect the acid–base status and energy budgets of marine organisms^4^,^5^,^6^. Predicted impacts of low pH are particularly severe for marine organisms with carbonate skeletons^7^,^8^,^9^, where calcification is significantly affected by a reduction of carbonate ion concentration^10^. However, some recent studies question the importance of the reduction of carbonate ion concentration^11^ and show the importance of other carbonate system species (i.e. HCO_3_^−^) during calcification^12^. Ocean acidification (OA) experiments consistently show a decline in the rate of calcification at CO_2_ and pH levels expected by the end of the century, although with considerable variability in the responses^7^,^8^,^9^. Calcifiers can respond to critical environmental stress, as low pH, through metabolic changes, development of protective membranes, and by enhancing the activity of proton and calcium pumps among others^5^. Most of these physiological processes are energy demanding, implying that the vulnerability of calcifiers to low pH seawater may depend on their energetic status^4^,^5^,^6^ and, therefore, be affected by food availability^13^. Hence, food availability may play a critical role in determining the resistance of calcifiers to OA^13^. Yet, 51% of the laboratory experiments testing responses of larvae, juveniles and adults of calcifying animals to OA included in a recent meta-analysis^9^ did not supply food to the animals during the experiments. These studies included experiments testing OA effects on mollusks^14^ and corals^15^, echinoderms^16^ and crustaceans^17^. This practice is contrary to proposed to best practices for experiments evaluating biological responses to OA^18^, which recommends that food supply mimicking those in natural conditions should be provided to ensure that the results of OA experiments and conclusions be applicable to natural systems and to avoid additional stress to specimens tested.

To date, the role of food supply on the response of marine calcifiers to OA has been assessed by a limited number of experiments. Accordingly, none of the meta-analyses conducted to-date did address the possible role of food supply on calcifiers^7^,^8^,^9^, including the meta-analysis^9^ used as a basis to evaluate the likelihood of impacts of OA in the recent AR5 assessment of the IPCC^19^. Here we test the hypothesis that food supply consistently confers resistance to marine calcifiers against OA. We do so based on a meta-analysis of reported results from experiments assessing the role of food availability on the growth and calcification of calcifying animals exposed to OA conditions.

## Results

**Figure 1:**
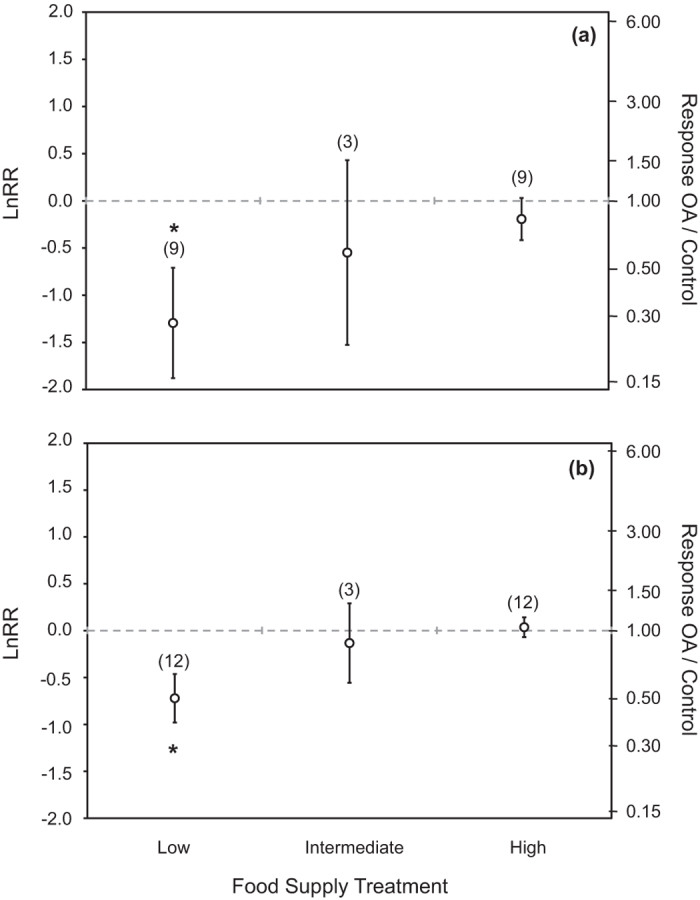
Mean effect size (LnRR) and response: control ratio of ocean acidification and food availability on calcification (**a**) and growth (**b**). Significance is determined when the 95% confidence interval does not cross zero. The number of experiments used to calculate the mean is included in parentheses. *denotes a significant effect.

A total of 12 published reports, most published very recently were found that experimentally assessed the effect of food supply on calcification and growth of marine calcifying animals, including echinoderms, crustaceans, molluscs and coral species under OA conditions. However, the limited number of studies available as yet precluded the examination of phylogenetic or ontogenetic differences, as our meta-analysis only encompassed 11 different species (Table S1). A total of 45 growth-responses observations and 35 calcification- responses observations were analyzed. Significant heterogeneity among effects sizes for calcification response (Q_T_ = 1056.50, d.f. = 34, *p* < 0.0001) and growth response (Q_T_ = 1471.42, d.f. = 44, *p* < 0.0001) were detected. The magnitude of the effects varied among food treatments for both growth (Q_M 1,43_ = 31.92, *p* < 0.0001) and calcification (Q_M 1,33_ = 13.81, *p* = 0.0002). In addition, there was a significant decrease in the effect size for calcification responses with increasing pH decline between control and acidification treatment under high food supply, providing evidence of a significant decline in calcification rates when pH was experimentally reduced by > 0.6 pH units under high food supply (Q_M 1,33_ = 6.46, *p* = 0.011, Table S2, Fig. S1). In contrast, the effect size for growth response was independent of the magnitude of the experimental reduction in pH (Q_M 1,43_ = 0.86, *p* = 0.353) for any food treatment (Table S2, Fig. S1). Food supply increased the resistance of marine calcifying animals to low pH for both processes (calcification and growth) examined (Fig. 1). OA conditions caused a significant reduction in calcification of 56.1 ± 10.8% relative to that at present pH when food was supplied at low level (Mann*-*Whitney *U*-test, *p* < 0.05) as opposed to statistically insignificant (Mann*-*Whitney *U*-test, *p* > 0.05) reductions of 27.5 ± 22.7% at intermediate food supply and a moderate 10.0 ± 11.7% decline at high food supply (Table 1). Increased levels of food supply also alleviated growth suppression under reduced pH, and no significant decline in growth was found when food supply was provided at intermediate or high concentrations (Table 1, Fig. 1). Whereas growth of calcifying organisms was reduced by 45.2 ± 5.3% (Mann*-*Whitney *U*-test, *p* < 0.05) when the animals were exposed to low pH and low levels of food supply, their growth rate tended to increase by 6.6 ± 6.5% (Mann*-*Whitney *U*-test, *p* > 0.05) under high food supply in acidified treatments (Fig. 1, Table 1).

## Discussion

Although to date only 12 studies have assessed the role of food supply on OA effects over calcifiers species, our results confirm that food supply reduces the impacts of experimental ocean acidification on the growth and calcification rates of a range of marine organisms including corals, molluscs, crustaceans and echinoderms. Whereas the number of assessments of the role of food supply on the performance of calcifiers under experimental ocean acidification conditions is still limited, the analysis suggests that marine calcifiers might be more resistant to ocean acidification than portrayed by existing assessments, based on meta-analysis including a large proportion of experiments were the organisms were not fed. In addition, our results suggest that forecasting the future of marine calcifiers requires consideration of both expected changes in carbonate chemistry and the productivity regimes of the ecosystems. Based on our analysis, the hypothesis that food supply confers marine calcifiers resistance against ocean acidification seems to hold. Although there seems to be an upper limit to this increased resistance as even organisms receiving high food supply may become vulnerable to OA when pH would decline by more than 0.6 units.

These findings are consistent with our understanding of the mechanisms allowing marine calcifiers to promote bio-calcification, a process mainly under biological control^20^, under the adverse conditions associated with OA. These mechanisms include the use of macromolecules (i.e. organic matrix proteins), proton and calcium pumps and the formation of membranes favoring aragonite or calcite deposition^21^,^22^,^23^,^24^. Some corals and foraminifera are able to increase pH at the site of calcification while some decapods, cephalopods and fishes can accumulate HCO_3_^−^ in extracellular fluids, thus buffering the negative impacts of hypercapnia and promoting calcification even under adverse conditions^5^. Bivalves lack the capacity to pump HCO_3_^−^ to increase pH within hemolymp or extrapallial fluid in order to avoid a decrease of pH at calcification sites^25^. Accordingly, the poor capacity of bivalves to regular their acid-base status has been inferred to render them particularly sensitive to the changes in the seawater carbon system involved in OA^26^. Nevertheless, bivalves exhibit a suite of mechanisms to cope with adverse pH environmental conditions to calcify, such as increased ammonia excretion, intracellular release of inorganic molecules and ions (i.e. CaCO_3_) and the presence of organic layers (i.e. periostracum or the shell organic matrix) guiding carbonate crystal deposition^27^,^28^,^29^,^30^,^31^. Although some of these mechanisms (i.e. capacity to increase pH at the site of calcification in corals) appears to be a particularly energy-efficient way to cope with pH variability^32^, the most of these processes are metabolically demanding, especially protein synthesis to generate the organic matrix of the shell^33^,^34^,^35^. Indeed, calcification processes have been reported to require 75% of the energy invested in somatic growth and 410% of that invested in reproduction by calcifying organisms such as echinoderms^36^. Food supply, thus, becomes significantly important in order to supply the energy required to support the metabolic processes facilitating bio-calcification under normal and specifically under adverse conditions associated with ocean acidification^5^.

The hypothesis that food supply confers marine calcifies resistance to OA is consistent with the current understanding of the energetic requirements of marine animals. It also would explain the apparent paradox that many upwelling areas around the world support some of the highest commercial fisheries of bivalves^37^,^38^ despite being frequently invaded by corrosive waters with pH even below the average pH in ocean surface waters expected by 2100^3^,^39^. Upwelling areas are characterized by very high primary production driven by the high-nutrients of upwelled waters^40^ and, hence, food supply, thereby conferring resistance to cope with their corrosive waters to calcifiers. However, the existence of lag of a few days between the upwelling of CO_2_–rich waters and peak primary productivity^41^ requires the presence of biological and evolutionary mechanisms (i.e. phenotypic plasticity) of calcifiers to cope with adverse conditions prior to increase food supply^5^,^42^,^43^. Hence, whereas high food supply certainly helps calcifiers support their characteristic high productivity in upwelling regions, this also requires local adaptations to cope with the adverse conditions the organisms experience immediately following upwelling events.

Our results show that caution should be taken to only base predictions of the response of calcifying organisms to future OA on pH or CO_2_ alone and that the role of concurrent changes in primary production should also be considered. For instance, the impact of OA on tropical and subtropical calcifiers, such as coral reefs, may be enhanced by parallel oligotrophication of the subtropical ocean driven by warming^44^. In contrast, over large spans of the Arctic Ocean, considered to be the ocean closest to experiencing corrosive conditions, warming and the loss of sea ice may lead to increased primary production^45^,^46^, possibly enhancing food supply, thus a possible increase in the resistance of Arctic calcifiers to OA. To date the expected response of Arctic pelagic calcifiers to OA is based solely on experiments with the pteropod Limacina^47^,^48^,^49^ conducted without offering food supply, possibly conducive to a perception of high vulnerability of these organisms compared to that they would experience under natural conditions and levels of food. Hence, concerted future trajectories in OA and food supply should be considered to formulate robust forecasts of the responses of marine calcifiers beyond speculations based on changes in one component alone.

Our analyses demonstrate the importance of OA experiments to adhere to best practices guidelines^18^ by providing adequate food levels when testing animals. The results point to the fact that failure to provide food to marine calcifiers can increase their vulnerability to OA in experimental assessments, which may yield inflated assessments of the impacts of OA on these organisms. Hence, the fact that more than half of the studies involving calcifying animals included in a recent meta-analysis^9^ did not supply food to the tested animals, may have lead to an overestimation of the impacts of OA on calcifying animals. This is of consequence as the results of that particular study were used as a basis to evaluate impacts of OA in the AR5 report of the IPCC^19^. Whereas the interaction between warming and OA has been addressed in the past^9^,^50^,^51^, models predicting the effect of OA on vulnerable marine calcifiers should also consider projected changes in food supply, which according to our results might act as main driver of the resistance of calcifiers to OA.

## Methods

### Bibliographic Search

We identified studies that measured any biological response to ocean acidification and included food supply treatments published to date. For the search, we used ISI web of science [v. 5.16.1©], the European Project on Ocean Acidification (EPOCA) blog (http://oceanacidification.wordpress.com/) and the updated bibliographic database from IAEA Ocean Acidification International Coordination Centre (OA-ICC)^52^, as well cross-references of the bibliographies of identified articles. We used the following keywords: “ocean acidification”, “food supply”, “food availability” and combinations of these. To date, only 12 experimental studies have been published which have recorded both the effects of OA and food supply.

### Data Extraction

We extracted the response of the investigated organism and/or process to the experimental OA treatments and the corresponding values of the control treatment. We used the experimental treatment with the highest pH and highest food supply level as control, and compared the results with any other treatment where pH was decreased and food supply was manipulated to different concentrations (high, intermediate and low food level). We followed the authors in ranking the food levels supplied. However, only five studies specifically justified the ecological relevance of the food levels supplied (see Table S1). In the case of low food treatments, this food treatment corresponded to a starvation regime in five of the 12 studies, while for the rest of the studies this treatment corresponded to food concentrations that reduced food considerably in comparison with that provided in the high food treatment. Provided the importance of food supply in the responses of marine calcifiers to OA, it is important that food supply be justified in relation to organismal requirements and the levels the organisms experience in the environment. When a single experiment reported several response variables that measured the same biological response, we included only one response per experiment to avoid pseudo-replication. When a biological response was measured repeatedly at different time intervals, we only considered the results for the final time point of exposure to experimental treatments. Calcification responses were primarily based on estimates of net calcification and skeleton weight while growth responses included estimates of change in biomass, length, cell volume and growth rates. There were insufficient data on other response variables such as metabolism, mortality and feeding rates to allow quantification of these effects. Data were extracted from tables, text and Pangaea® database. To obtain data from graphics we used the software GraphClick (version 3.0) (Neuchatel, Switzerland).

### Data Analysis

Log-transformed response ratio (LnRR), which is the ratio of the mean effect in the acidification treatment to the mean effect in a control group^53^ was calculated. A log-transformed response ratio of zero is interpreted as the experimental treatment having no effect on the response variable, while a positive value indicates a positive effect and a negative value indicates a negative effect. The statistical significance of mean effect sizes is based on bias-corrected bootstrapped 95% confidence intervals. When these 95% confidence intervals do not overlap zero, the effect size is considered significant (α = 0.05). In order to determine if there was variation of effect sizes magnitude among studies (heterogeneity) for both growth and calcification responses a Q_T_ (α = 0.05) statistic test was performed^54^. The variation in effect sizes among food treatments and ΔpH (reduction from control conditions) for all growth and calcification observations were tested using a random effects meta-analysis. Lastly, the relationship between differences in effects with the experimental pH change (ΔpH) was tested using linear regression for each food treatment. Additionally, effects of OA for each food supply treatment were calculated as a percentage related to control conditions (see above). Statistical differences in calcification and growth between control and experimental treatments were tested using a Mann-Whitney test. All of the analyses were performed using JMP software (Version 9.0.1) and R routines (metafor package) (R Development Core Team 2009).

## Additional Information

**How to cite this article**: Ramajo, L. *et al*. Food supply confers calcifiers resistance to ocean acidification. *Sci. Rep.* **6**, 19374; doi: 10.1038/srep19374 (2016).

## Supplementary Material

Supplementary Information

## Figures and Tables

**Table 1:** Growth and calcification under acidified conditions relative to present pH and food supply levels.

Biological Variable	Food Supply Treatment		
	Low	Intermediate	High
Calcification	43.9 ± 10.8%^(*)^	72.5 ± 22.7%^(NS)^	90.0 ± 11.7%^(NS)^
Growth	54.8 ± 5.3%^(*)^	92.0 ± 15.0%^(NS)^	106.6 ± 6.5%^(NS)^

Data are mean ± SE percent relative to control. H_o_ no difference from control (percent = 100%): NS = *p* > 0.05, * *p* < 0.05.
