# Supplementary material for: Food supply confers calcifiers resistance to ocean acidification
Source: Sci Rep. 2016 Jan 18;6:19374. doi: 10.1038/srep19374 (PMC4726000; doi:10.1038/srep19374)

SUPPLEMENTARY MATERIAL

Food supply confers calcifiers resistance to ocean acidification

Laura Ramajo*, Elia Pérez-León, Iris E. Hendriks, Núria Marbà, Dorte Krause-Jensen, Mikael K. Sejr, Martin E. Blicher, Nelson A. Lagos, Ylva S. Olsen, and Carlos M. Duarte

Table S1. Studies, taxa, species, life stage, experimental pCO2 and food levels used in the experimental studies included in the meta-analysis.

Reference[1]	Taxa	Species	Life Stage	*pCO*$_2$ Treatments Control	OA	Food Treatments
Edmunds (2011)	Coral	*Porites spp.*	Juvenile	41.6 (Pa)	81.5 (Pa)	HF: Fed (*ad libitum*)<br>LF: Unfed (starvation)
Melzner *et al.,* (2011)	Mollusc	*Mytilus edulis*	Juvenile	390 (ppm)	1120, 2400 and 4000 (ppm)	HF[4]: 1600–2000 cells mL^{-1}<br>LF: 310– 350 cells mL^{-1}
Thomsen *et al.,* (2013)	Mollusc	*Mytilus edulis*	Juvenile	380 (µatm)	1120, 2400 and 4000 (µatm)	10 (HF) : 5 (IF) : 1(LF)
Hettinger *et al.,* (2013)	Mollusc	*Ostrea lurida*	Larvae	500 (µatm)	1000 (µatm)	50 (HF)[4] : 25 (IF) : 5(LF)
Crook *et al.,* (2013)	Coral	*Balanophyllia elegans*	Larvae	410 (µatm)	770 and 1220 (µatm)	HF: Every 3 days<br>LF: Once during all experiment
Comeau *et al.,* (2013)	Coral	*Porites rus*	Adult	400 (µatm)	700 (µatm)	HF[4]: Fed (*ad libitum*)<br>LF: Unfed (starvation)
Drenkard *et al.,* (2013)	Coral	*Favia fragum*	Juvenile	421 (µatm)	1311 (µatm)	HF[4]: Similar to environment<br>LF: Unfed (starving)
Pansch *et al.,* (2014)	Crustacean	*Amphibalanus improvisus*	Juvenile/ Adult	Ambient (atmospheric air)	1120 and 4000 (ppm)[2]<br>977 and 3000 (ppm)[3]	5(HF) : 1(LF)
Oddvarstdotter (2014)	Crustacean	*Calanus finmarchicus*	Larvae	380 (ppm)	2080 (ppm)	HF: 600 µgC L^{-1}<br>LF: 150 µgC L^{-1}
Pan *et al.,* (2015)	Echinoderm	*Strongylocentrotus purpuratus*	Larvae	Ambient (atmospheric air)	800 (µatm)	HF: Fed (*ad libitum)*<br>LF: Unfed
Towle *et al.,* (2015)	Coral	*Acropora cervicornis*	Adult	390 (ppm)	800 (ppm)	HF: Fed (*ad libitum*)<br>LF: Unfed (starvation)
Ramajo *et al.,* (submitted)	Mollusc	*Argopecten purpuratus*	Juvenile	450 (µatm)	1400 (µatm)	HF[4]: 5% ind DW d^{-1}<br>IF: 2% ind DW d^{-1}<br>LF: 0.1% ind DW d^{-1}

[2]Kiel population [3]Tjärnö population [4]similar to environment or ecologically-relevant

[1]References

Edmunds PJ (2011). Limnology and Oceanography 56(6): 2402-2410.

Melzner et al. (2011). PlosOne 6(9): e24223.

Thomsen et al. (2013). Global Change Biology 19: 1017-1027.

Hettinger et al. (2013). Biogeosciences 10: 6629-6638.

Crook et al. (2013). Biogesosciences 10: 7599-7608.

Comeau et al. (2013). Marine Biology 160: 1127-1134.

Drenkard et al. (2013). Coral Reefs 32: 727-735.

Pansch et al. (2014). Global Change Biology 20: 765-777

Oddvarstdotter (2014). Master Thesis. Norges teknisk-naturvitenskapelige universitet. (http://urn.kb.se/resolve?urn=urn:nbn:no:ntnu:diva-25205)

Pan et al. (2015). PNAS 112 (15): 4696–4701,

Towle et al. (2015). PlosOne. doi:10.1371/journal.pone.0123394.

Ramajo et al. (accepted). Biomineralization changes with food supply confer juvenile scallops (*Argopecten purpuratus*) resistance to ocean acidification. Global Change Biology

Table S2. Effect of the magnitude of pH reduction (ΔpH) on effect sizes (LnRR) on calcification and growth responses for high (HF), intermediate (IF) and low (LF) food treatments. *denotes significant effect ($\alpha = 0.05$)

Response	Food	df	Slope	P-value
Calcification	HF	13	-1.026	0.019*
	IF	3	-2.996	0.068
	LF	13	-2.197	0.067
Growth	HF	18	-0.386	0.066
	IF	3	-1.065	0.189
	LF	18	-0.111	0.837

Figure S1. LnRR estimates for calcification (a) and growth (b) responses with individual observations plotted against ΔpH (pH reduction from control conditions). The solid (significant) and dotted (not significant) lines show the fitted regression of effect sizes versus ΔpH for each food treatment.

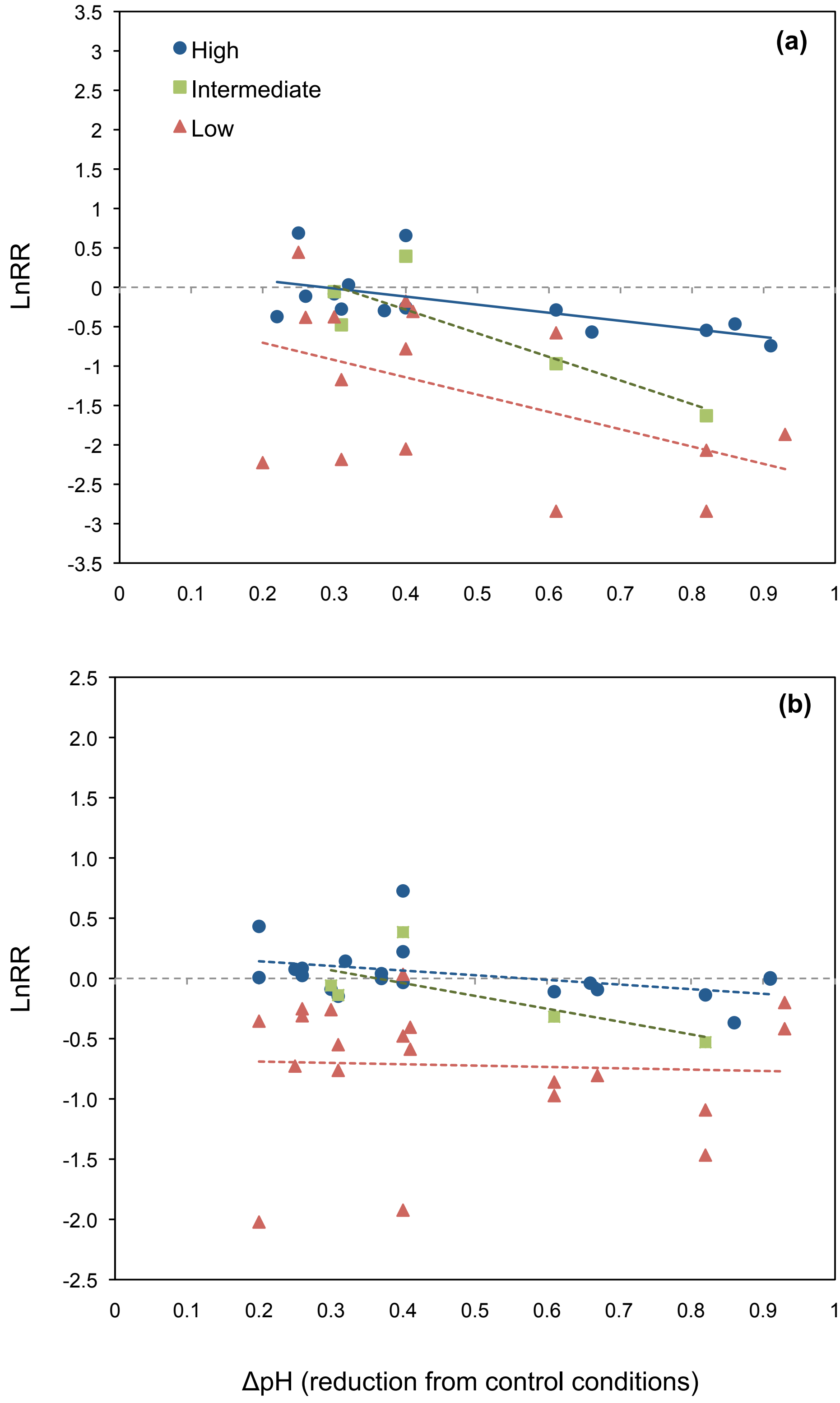

Supplement: Supplementary Information [file srep19374-s1.pdf]
